# Brain structural plasticity with spaceflight

**DOI:** 10.1038/s41526-016-0001-9

**Published:** 2016-12-19

**Authors:** Vincent Koppelmans, Jacob J Bloomberg, Ajitkumar P Mulavara, Rachael D Seidler

**Affiliations:** 1 School of Kinesiology, University of Michigan, 401 Washtenaw Ave., Ann Arbor, MI 48109-2214 USA; 2 NASA Johnson Space Center, Houston, TX 77058 USA; 3 KBRwyle, Houston, TX 77058 USA; 4 Department of Psychology, University of Michigan, Ann Arbor, MI 48109 USA

## Abstract

Humans undergo extensive sensorimotor adaptation during spaceflight due to altered vestibular inputs and body unloading. No studies have yet evaluated the effects of spaceflight on human brain structure despite the fact that recently reported optic nerve structural changes are hypothesized to occur due to increased intracranial pressure occurring with microgravity. This is the first report on human brain structural changes with spaceflight. We evaluated retrospective longitudinal T2-weighted MRI scans and balance data from 27 astronauts (thirteen ~2-week shuttle crew members and fourteen ~6-month International Space Station crew members) to determine spaceflight effects on brain structure, and whether any pre to postflight brain changes are associated with balance changes. Data were obtained from the NASA Lifetime Surveillance of Astronaut Health. Brain scans were segmented into gray matter maps and normalized into MNI space using a stepwise approach through subject specific templates. Non-parametric permutation testing was used to analyze pre to postflight volumetric gray matter changes. We found extensive volumetric gray matter decreases, including large areas covering the temporal and frontal poles and around the orbits. This effect was larger in International Space Station versus shuttle crew members in some regions. There were bilateral focal gray matter increases within the medial primary somatosensory and motor cortex; i.e., the cerebral areas where the lower limbs are represented. These intriguing findings are observed in a retrospective data set; future prospective studies should probe the underlying mechanisms and behavioral consequences.

## Introduction

Humans undergo extensive sensorimotor adaptation during spaceflight due to altered vestibular inputs and unloading of the body. No studies have yet evaluated the effects of spaceflight on human brain structure. This is despite the fact that recently reported optic nerve structural changes are hypothesized to occur due to increased intracranial pressure occurring with fluid shifts towards the head in microgravity.^1^ NASA has recently laid out plans for remote space exploration, including potential human travel to Mars.^2^ Microgravity has negative effects on physiological systems, including muscle and bone mass loss, which are targeted with exercise and pharmacological countermeasures. Long-term head down tilt bed rest, an established spaceflight analog, leads to a superior–posterior shift of the brain within the skull,^3^ suggesting that microgravity exposure may lead to similar results.

The untoward effects of spaceflight on sensorimotor function have been well-studied, including postflight impairments in posture control^4,5^ and locomotion,^4,6–8^ as well as inflight spatial disorientation,^9^ reduced mass discrimination^10^ and increased manual tracking errors under cognitive load.^11,12^ Astronauts gradually adapt their sensorimotor processing inflight in response to body unloading and altered vestibular inputs. This adapted sensorimotor state is then inappropriate upon return to Earth’s gravitational environment, with astronauts exhibiting slow re-adaptation over days and even weeks postflight.^8^

Based on the extensive literature documenting experience-dependent brain plasticity,^13,14^ one might predict that neuroplasticity occurs in sensorimotor cortical regions with spaceflight. Indeed, experiments conducted with rodents have reported changes with spaceflight such as alterations in the distribution of axonal terminal type in the somatosensory cortex^15^ and degeneration of Purkinje cell dendrites.^16^ A recent human case study reported increases in motor cortex-cerebellar functional connectivity with spaceflight.^17^ In addition to specific sensorimotor structural plasticity, it is also possible that the accompanying cephalic fluid redistribution, sleep loss, and other stressors of spaceflight would result in nonspecific structural brain atrophy or edema. Evaluating the extent and nature of human brain structural changes with spaceflight and how this relates to sensorimotor performance is critical given the increasing duration of human spaceflights including advanced plans for remote explorations to Mars.^2^ Furthermore, it provides an opportunity to study brain changes occurring with sensorimotor adaptation on a much longer timescale than has ever been investigated. Previous work using brain imaging and stimulation approaches has found activity in a right lateralized frontoparietal network as well as motor corticocerebellar regions that is associated with visuomotor adaptation^18–20^ but study protocols have only examined brain changes occurring with a handful of practice sessions. Here, we evaluated retrospective longitudinal T2-weighted MRI scans and balance performance data from 27 astronauts obtained from the NASA Lifetime Surveillance of Astronaut Health to determine the effects of spaceflight on human brain structure, and whether any changes are associated with the magnitude of balance decrements observed from pre to postflight.

## Method

### Study design

The current study is a retrospective follow-up study. T2-weighted MRI scans of 27 astronauts were obtained from the NASA Lifetime Surveillance of Astronaut Health. Because of the retrospective nature of this study, not all MR images were collected using the same protocol.

### Participants

We included data from 27 astronauts of whom 13 completed a space shuttle mission (~2 weeks) and 14 completed a mission to the International Space Station (ISS) (~6 months). Demographic information is presented in Table 1. The astronauts’ age ranged from ~40 to ~60 years (mean = 48.0, sd = 3.6), while their mission duration ranged from 12 to less than 200 days. Prior spaceflight experience in these astronauts ranged from 0 (two astronauts) to >300 days. The MRI and neurosensory data were collected for mission-related medical monitoring. All astronauts in this study provided written informed consent for this retrospective analysis.

**Table 1:** Demographics

	Shuttle (*n* = 13)		ISS (*n* = 14)			
	Mean	Sd	Mean	Sd	*F*(1, 27)	*p*-value
Age (years)	47.2	1.0	48.8	1.0	1.40	0.25
Mission duration (days)	13.7	3.4	162.0	3.3	975.22	<0.001
Days in space pre-flight	88.5	24.2	40.6	23.3	8.82	0.007

*ISS* International space station

### Balance control

Balance control was measured using the Sensory Organization Tests (SOTs) provided by the EquiTest System platform (NeuroCom, Clackamas, OR).^21^ Out of 27 astronauts there were 21 subjects with complete SOT assessment at both pre and postflight assessments where the postflight measurement was collected within the first two days postflight. We report data from all trials that were conducted with sway-referenced support surface intended to disrupt somatosensory feedback and with eyes closed (SOT5); this test reflects how well vestibular input could be utilized to maintain balance.

### Image acquisition

Within the group of 27 astronauts either (1) low resolution (*n* = 10) or (2) high-resolution (*n* = 17) pre- and postflight image pairs were acquired:

1. Low-resolution scans were either sagittal (*n* = 8) or axial (*n* = 2) acquisitions. In both cases MRI was performed on a 3T Philips Intera MRI scanner with an 8-channel head coil. Sagittal T2-weighted sensitivity encoding (SENSE) images had the following parameters (TR = 6.1 s, TE = 80 ms, flip angle = 90°, number of signal averages (NSA) = 1, field of view (FOV) = 240 × 240 mm, slice thickness = 3.0 mm (no slice gap), 50 sagittal slices, matrix = 512 × 512, and voxel size = 3.00 × 0.47 × 0.47 = 0.66 mm^3^). Identical parameters were used for pre- and postflight data collection, except for one out of these ten subjects for whom post data was collected with a slightly larger FOV (i.e., 256 × 256 mm) resulting in an in-plane voxel size of 0.50 × 0.50 mm. Axial T2-weighted SENSE images had the following parameters (TR = 3.6 s, TE = 80 ms, flip angle = 90°, NSA = 2, FOV = 240 × 240 mm, slice thickness = 4.0 mm (1mm slice gap), 32 axial slices, matrix = 512 × 512, and voxel size = 0.47 × 0.47 × 5.00 = 1.10 mm^3^).
2. All high-resolution T2 scans were obtained using a 3T SIEMENS Verio scanner applying a sagittal 3D TSE (turbo spin echo) SPACE (sampling perfection with application optimized contrasts by using different flip angle evolutions) sequence with the following scan parameters: parameters (TR = 3.2 s, TE = 409 ms, flip angle = 120°, NSA = 1, FOV = 250 × 250 mm, slice thickness = 1.0 mm (no slice gap), 176 sagittal slices, matrix = 512 × 512, and voxel size = 1.00 × 0.49 × 0.49 = 0.24 mm^3^).

Preflight scans were collected at a median of 194 days before launch (range = 18–627). Postflight scans were collected at a median of 6 days after return (range = 1–20).

### Image processing

Longitudinal voxel-based morphometry and region of interest (ROI) analyses (see below, under “Sensorimotor Regions of Interest”) were used to detect significant changes in brain gray matter volume from preflight to postflight. Voxel-based morphometry involves voxel-wise comparison of probabilistic gray matter maps that have been transformed to the same stereotactic space.^22–24^ The following software packages were used for image processing: Advanced Normalization Tools (ANTs) version 1.9.x, FMRIB Software Library (FSL) version 5.0.8, Statistical Parametric Mapping (SPM) 8 v6313, SPM 12 v6470, and MATLAB 8.3.0.532 (R2014a).

### Preprocessing

Image intensity non-uniformity correction was applied to all T2 images within a subject specific brain mask using N4ITK with a shrink factor of 2 and 80, 60, and 40 iterations at each level of resolution.^25^ The brain masks were created using FSL’s Brain Extraction Tool^26^ with robust brain center estimation.

### Segmentation

Bias field corrected images were segmented into 6 probabilistic tissue classes (i.e., gray matter, white matter, cerebrospinal fluid, bone, fat, and air) using unified segmentation with a sampling distance of 1 under SPM 12.^27,28^ The unified segmentation algorithm uses a prior probability map per tissue class and voxel intensity to attribute the a posteriori probability of each voxel belonging to a tissue class. Bias regularization was set to ten and the bias full width at half maximum (FWHM) cutoff was set to 150 because of our initial non-uniformity correction. Segmentation quality of the high-resolution and low-resolution images (see Supplementary Fig. 1) was satisfactory.

### Normalization

High-resolution images (i.e., 0.49 × 0.49 × 1.00 mm) were down sampled to 0.98 × 0.98 × 1.00 mm to reduce memory costs and speed up the normalization process.^29^ We used a stepwise approach to transform the preflight and postflight gray matter maps of each subject into MNI space. This method first registers all bias field corrected T2 images to an initial template using six degrees of freedom. Transformation parameters were stored in the header of the image to avoid rounding. Separate initial template images were created for the high-resolution image pairs and the low-resolution image pairs to fit their initial resolutions. These templates were constructed by rigid body registration of the T2 MNI ICBM152 nonlinear symmetric image^30^ to the average of the non-transformed data in our sample that were acquired using a sagittal sequence (i.e., eight out of ten astronauts for the low-resolution images and 17 astronauts for the high-resolution image). An initial subject specific template was created from each co-registered pair of preflight and postflight scans by averaging the two images and subsequently smoothing the resulting image with a Gaussian kernel of 1 mm. For further normalization steps we selected ANTs because it has proven to be superior in normalization than other algorithms and offers readily available scripts to create templates and combine warp fields.^31^ From ANTs, we used *buildtemplateparallel* with the initial subject specific template as reference image with probability mapping as similarity metric and symmetric normalization as transformation model to create a final subject specific template. After that, we calculated the warp from the single subject template to the T2 MNI ICBM152 nonlinear symmetric image (2009a)^30^ using ANTs with cross correlation as similarity metric and symmetric normalization as transformation model. For each subject, for each time point the non-linear warp field from subject space to the subject specific template and the non-linear warp field from the subject specific template to the MNI template were combined into one flow field. From this flow field we obtained the Jacobian determinant image using ANTs’ *CreateJacobianDeterminantImage*. The Jacobian determinant encodes local expansion/shrinkage for each voxel in the image. The gray matter map of each subject, for each time point was then warped to MNI space using the combined affine and non-linear transformations that we obtained from brining the subject specific and time point specific T2 image into MNI space. Subsequently, these normalized images were modulated by multiplying them with their Jacobian determinant image to preserve the amount of gray matter volume that was present in the untransformed image. Finally, the modulated warped images were smoothed with a Gaussian kernel of 8 mm FWHM to increase the signal to noise ratio.

### Sensorimotor regions of interest

A spherical ROI around MNI coordinate = 42, −24, 18 with a diameter of 5 mm (33 mm^3^) was used to mask out gray matter volume of the smoothed modulated gray matter images in MNI space as a proxy of the right vestibular cortex. This voxel coordinate represents the center of operculum parietale 2 (i.e., the homolog of the parietoinsular vestibular cortex in nonhuman primates).^32, 33^ Volumes of the precentral gyrus, postcentral gyrus, and paracentral gyrus were also obtained per subject per time point by masking out the gray matter volume in these regions from the smoothed modulated gray matter images in MNI space. The masks were obtained from the T1 MNI ICBM152 using Freesurfer.^34^ Finally, we tested for changes in global tissue volume of gray matter, white matter, and cerebrospinal fluid.

### Analysis

Voxel-wise nonparametric sign-flip and permutation based one-sample *t*-tests on pre-to-post GM difference maps with 15,000 random permutations and threshold-free cluster enhancement (TFCE)^35^ implemented in FSL’s randomize^36^ were used to test (1) if there were local gray matter increases or decreases as a function of spaceflight, (2) if there was a difference in GM changes between astronauts who completed a shuttle mission versus an ISS mission, and (3) if focal GM volume obtained at the preflight scan was associated with previous flight experience in days (i.e., a cross-sectional analysis). Non-parametric permutation tests with 15,000 random permutations and TFCE were used to analyze the association between local changes in GM volume and changes in balance control. Analysis of this association was conducted at the whole brain level as well as restricted to those locations in which we observed significant changes in GM volume from preflight to postflight and was performed in the whole group as well as stratified for mission type (ISS versus Shuttle). Because we only included the non-linear transformation in the Jacobian determinant image and not the affine transformations, the GM maps were already scaled for head size and subsequent adjustment for head size was unnecessary. All voxel-wise analyses were adjusted for multiple comparisons by applying a family-wise error correction (*p* < 0.1).

Changes in regional volume and balance control were analyzed using linear-mixed models with subject as random intercept and restricted maximum likelihood (REML) as maximum likelihood estimation because REML is less sensitive to small sample bias than traditional maximum likelihood estimation.^37^ Regional volumes were obtained from gray matter maps that were scaled for head size and thereby adjusted for total intracranial volume. Any significant changes in regional GM volume were correlated with changes in balance performance using Spearman’s rank correlation test. Alpha levels were set at 0.05 for all analyses. Stata SE was used for all analyses (StataCorp. 2013. Stata Statistical Software: Release 13.1. College Station, TX: StataCorp LP).

## Results

Astronauts performed significantly worse on measures of standing balance performance postflight compared to preflight (see Table 2).

**Table 2:** Global and regional volumetric brain changes and balance control changes from pre-flight to post-flight

					95 % CI		
	*N*	Average pre-flight	Difference post-flight	SE of the difference	Lower	Upper	*p*-value
SOT5 (%)	21	84.7	−13.4	3.3	−19.8	−7.0	<0.001
SOT5-HM (%)	21	77.9	−43.1	5.0	−52.8	−33.2	<0.001
Global GM (ml)	27	611.4	−5.7	5.9	−17.3	5.9	0.34
Global WM (ml)	27	581.3	1.5	5.9	−10.1	13.1	0.80
Global CSF (ml)	27	302.7	4.2	4.5	−4.6	12.9	0.35
ICV (ml)	27	1494.9	0.8	3.0	−5.0	6.6	0.78
Pre CG (ml)	27	6.31	0.02	0.11	−0.19	0.23	0.86
Post CG (ml)	27	4.01	<0.01	0.07	−0.13	0.12	0.94
Para CG (ml)	27	1.60	0.07	0.03	0.02	0.13	0.012
Vestibular cortex ROI (μl)	27	629.5	6.9	8.5	−23.5	9.7	0.41

Volumetrics were adjusted for total intracranial volume (tICV)

*CI* confidence interval, *SE* standard error, *GM* gray matter, *WM* white matter, *CSF* cerebro spinal fluid, *CG* central gyrus, *ROI* region of interest, *SOT5-HM* sensory organization test 5 with head movement

**Fig. 1:**
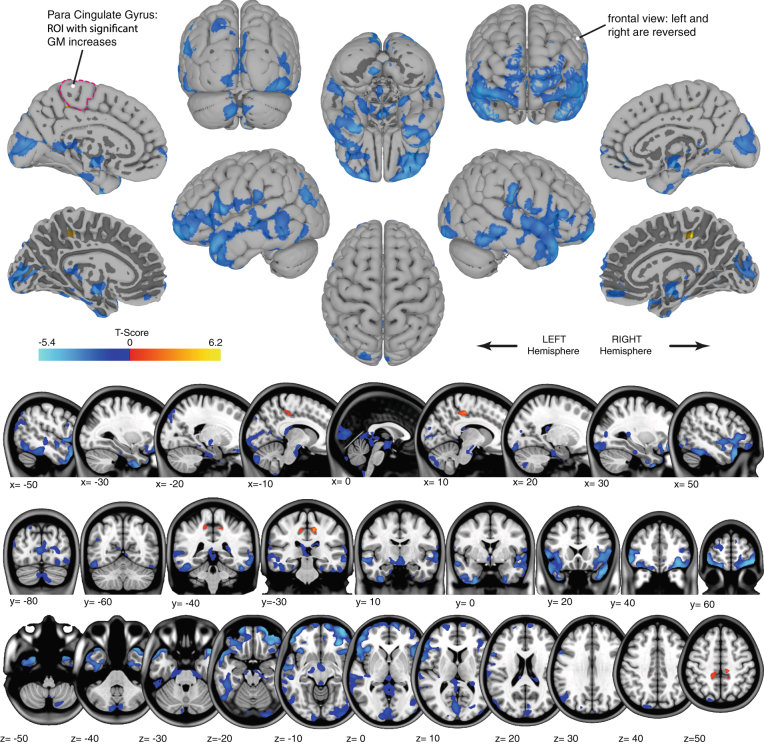
*Gray* matter changes as a function of spaceflight. *Red-to-yellow* gradients show regions with significant *gray* matter volume increase. *Blue* gradients show regions with significant *gray* matter decreases. Results are displayed on *top* of the pial surface of the ICBM MNI brain (surface view; *top*) or overlaid on the ICBM MNI T1 image (*bottom*). For the medial surface views, additional surface images that show the border of the *white* and *gray* matter are provided (marked with “*”) to show the *gray* matter changes that are present in deeper sulcal regions. The *right side* of the image corresponds with the *right side* of the brain, unless mentioned otherwise. The para cingulate gyrus that shows significant overall *gray* matter increases from pre- to post-flight is outlined on one of the surface images

**Fig. 2:**
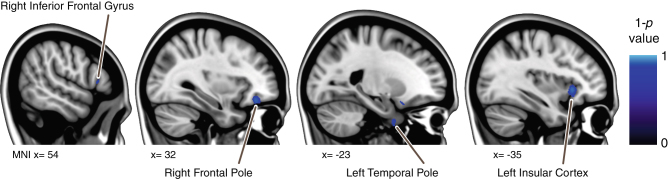
Focal differences in *gray* matter volume changes between shuttle astronauts and ISS astronauts. *Blue* gradients show regions in which ISS astronauts had significantly larger decreases in *gray* matter than shuttle astronauts from pre to post-flight. Results are overlaid on the MNI152 brain

Family wise error corrected analysis of the gray matter maps revealed significant widespread volumetric decreases as well as more localized increases. The GM decreases were distributed broadly around: the temporal and frontal poles, in lateral inferior temporal and frontal areas, around the orbits, and in bilateral medial parts of Crus II of the cerebellum. The GM increases overlapped the precentral gyrus, postcentral gyrus, the posterior cingulate gyrus, and precuneus cortex (see Fig. 1). We observed some regions in which GM decreases were significantly larger in astronauts who completed an ISS mission than in those who completed a shuttle mission (see Fig. 2). Furthermore, no association was observed between focal GM volume measured preflight and days of previous spaceflight experience. No correlations were observed between pre to postflight changes in focal GM volume and corresponding changes in standing balance performance.

Given that altered vestibular function and central re-interpretation of vestibular inputs and body unloading underlies much of the spaceflight-induced sensorimotor disturbances,^38^ we evaluated potential volumetric GM changes in the precentral gyrus, postcentral gyrus, paracentral gyrus, and vestibular cortex. Except for the paracentral gyrus, no significant changes were observed in these regions (see Table 2). Additionally, no significant changes were observed in global GM, white matter, CSF, and total intracranial volume (see Table 2). No correlations were observed between pre to postflight changes in regional GM volume and changes in standing balance performance.

**Fig. 3:**
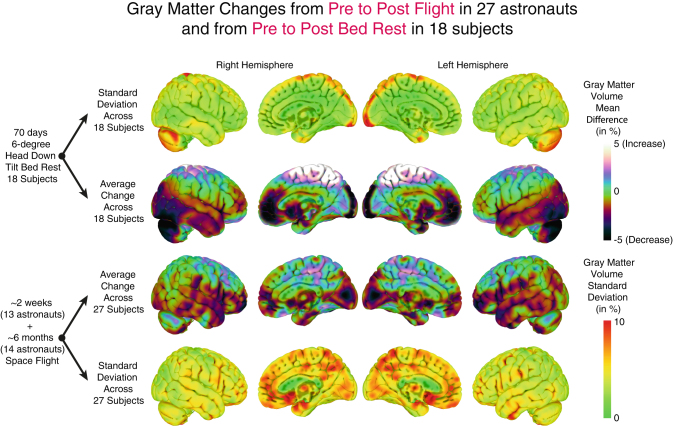
Qualitative comparison of focal *gray* matter changes with bed rest and spaceflight. Images on the *two middle rows* show average focal *gray* matter changes from pre to post-bed rest [18 subjects] and from pre to post-spaceflight [27 subjects] plotted on the ICBM MNI brain. *Red-to-yellow* gradients indicate *gray* matter increases whereas *blue* gradients indicate *gray* matter decreases. The *top* and *bottom rows* show the standard deviation of focal *gray* matter changes in these samples. This overview is intended for qualitative comparison. No statistical tests were run because of differences in pre-to-post intervals, scan sequence and parameters, and demographic differences

To aid in the interpretation of the here reported focal gray matter changes from preflight to postflight we have made a qualitative comparison of the average focal gray matter changes in these astronauts with the average gray matter changes that we observed in a group of 18 subjects who participated in our microgravity-analog head down tilt bed rest study.^39,40^ No statistical tests were run because of differences in pre-to-post intervals, scan sequence and parameters, and demographic differences. Figure 3 presents the mean and standard deviation of pre- to post-intervention changes for both long duration head down tilt bed rest and spaceflight. There are extensive similarities between the two, with the pattern of increases and decreases shifted somewhat posteriorly in the bed rest subjects relative to flight. Between subject variability is generally higher in the flight than bed rest subjects, potentially due to variability in mission duration and scan quality. A notable exception to these similarities in mean change and variability is observed in the cerebellum. To provide insight into the association between GM changes and CSF changes, we have plotted areas in which the average change over all subjects in CSF and GM from pre to postflight was larger than 1 %. We then binarized these areas and plotted them on a single brain to show where GM increases overlapped with CSF increases and vice versa (see Supplementary Fig. 1).

## Discussion

We observed significant increases and decreases in GM volume from pre to postflight in a group of 27 astronauts. The widespread gray matter decreases were located around the frontal and temporal poles and the orbits. We also observed small but localized gray matter increases in sensorimotor brain regions. There were also significant increases in the paracentral gyrus in our ROI analysis that we did not pick up in the voxel-wise analysis. This discrepancy can be explained by the fact that ROI analyses can be more sensitive to pick up small effects over a larger region whereas voxel-wise analysis can be more sensitive to pick up large focal changes, which is one of the reasons why we chose to apply both approaches.

The GM decreases that we observed could reflect focal changes in CSF due to the probabilistic nature of the segmentation algorithm.^28^ The changes in GM volume overlapped somewhat with changes in CSF volume in our sample, although there were also widespread differences. This suggests that the GM changes that we observe may be related to CSF redistribution. Further evidence for this idea comes from patients suffering from idiopathic normal pressure hydrocephalus (iNPH^41,42^). Several VBM studies in iNPH patients revealed increases in CSF and decreases in GM (and vice versa) in regions very similar to ours.^43–45^ Our results are further in line with a spaceflight analog study that used T2-images to study regional changes in CSF volume as a function of bed rest; the authors reported CSF decreases in a predefined location in the posterior parietal subarachnoidal space.^46^ However, inferior frontal regions in which we observed gray matter decreases were not examined in the previous study, which precludes further comparison. Hence, in general our results largely parallel findings from long duration head down tilt bed rest studies in terms of the distribution of GM changes that were attributed to an upward shift of the brain’s center of mass.^3,47^ A qualitative comparison of our data with bed rest data also showed differences in the regions in which we observed GM changes, such as in the cerebellum. This suggests that bed rest and spaceflight have some similar but also unique effects on the brain. Interestingly, GM volumetric decreases were larger in certain regions including the insular cortex in astronauts who completed an ISS mission compared to shuttle flight. Despite the lack of correlation between days previously spent in space and focal GM volume maps obtained preflight this finding suggests a dose-response effect for GM volumetric decreases with spaceflight.

It is possible that the small but localized gray matter increases we observed in sensorimotor brain regions parallel what has been reported in studies of neuroplasticity occurring with extended practice.^48,49^ For instance, it has been observed that one week of adaptation training results in gray matter volumetric changes in the motor cortex which furthermore predicted retention of sensorimotor adaptation over days.^50^ In the current study, crew members were exposed to the adaptive microgravity stimulus continuously throughout their spaceflight. Typically studies of sensorimotor adaptation have participants practice for less than an hour at a time,^51^ or examine adaptation interacting with injury or disease.^52^ In contrast, our findings may represent a greater reserve in the maximum capacity for neuroplasticity in the healthy human brain and suggests the utility of enhancing neuroplasticity to adapt to change in sensorimotor environments as potential countermeasure application in general.^53^ It is particularly compelling that the GM increases were restricted to medial sensorimotor regions which represent the lower limbs. Lower limb muscles are extensively active on Earth to counteract gravity, and exhibit the largest morphological changes with spaceflight.^54^ Increasing GM volume in the brain regions which process lower limb somatosensory inputs and motor control may reflect an attempt by the system to increase input sensitivity of representations and adaptation of lower limb control to the microgravity environment. Indeed a bed rest study has reported that corticospinal excitability is increased as a function of long term unloading.^3^ Sensorimotor novelty and practice have been associated with positive brain plasticity and protection of neural tissue,^55,56^ particularly in sensorimotor brain regions in relation to spaceflight,^15,16^ and this may be what led to the sensorimotor structural brain plasticity we observed here. Gray matter increases observed with MRI can reflect different neuroplastic processes that could take place simultaneously such as gray matter plasticity. Although neurogenesis in the adult brain is limited to certain regions (e.g., the dentate gyrus of the hippocampus, the subventricular zone of the lateral ventricle, and the olfactory bulb) other neuroplastic changes including axon sprouting, dendritic branching and synaptogenesis, changes in glial number and morphology, and angiogenesis occur over the adult life course.^14^

There are several factors that could have affected our results that are inherent to the retrospective nature of our study. Low image resolution, considerable intervals between the launch and pre and postflight MRI assessments, and previous flight experience may have masked some pre to postflight changes. Additionally, differential time intervals between preflight and postflight MRI and SOT assessments may have negatively impacted our ability to detect a true correlation between brain changes and balance performance. Also, even though we did not observe associations between balance and GM changes, these structural changes may be associated with other motor disabilities that occur frequently in astronauts postflight. A controlled longitudinal prospective study looking into brain changes and changes in balance, gait, and fine motor skill as a function of spaceflight is currently underway.^39^ Ideally, such a study would include free-water analysis of diffusion tensor MRI to assess fluid shifts, considering the high sensitivity of this method to changes in interstitial fluid.^57,58^ Furthermore, since preflight measures were obtained generally a long period before flight and therefore, the changes that we observed could partially reflect the intensive training that astronauts complete in preparation for their missions. This effect may be tempered because this preflight training typically starts at a minimum of 2 years before launch. Preflight MRI assessment for 20 out of 27 subjects was completed within one year before launch and therefore, the effects of this training on brain structure may have already been largely accounted for in the preflight measure. Despite all of these shortcomings, we still observed widespread GM decreases and increases occurring with spaceflight that resemble the pattern of GM changes that we observed in a microgravity analog bed rest study (see Fig. 3).^39,40^ This is the first documentation of structural brain changes after spaceflight in human subjects. Ongoing prospective controlled studies will be important to refine our findings, to understand the mechanisms, and to characterize both the performance relevance and recovery timecourse for these changes. Thus, the potential underlying mechanisms of these GM changes should be considered speculative due to the retrospective and heterogenic nature of our data.

In sum, our data show structural brain changes occurring with spaceflight that could be related to cephalad fluid shifts or neuroplasticity. Moreover, we found evidence of dose-response effects for GM decreases. More carefully controlled prospective studies may shed further light on these changes and their relation to behavioral performance.

## Electronic supplementary material

Supplementary Figure 1

Supplementary Figure 2
