# Erratum: Brain structural plasticity with spaceflight

**DOI:** 10.1038/s41526-017-0012-1

**Published:** 2017-11-27

**Authors:** Vincent Koppelmans, Jacob J Bloomberg, Ajitkumar P Mulavara, Rachael D Seidler

**Affiliations:** 1 School of Kinesiology, University of Michigan, 401 Washtenaw Ave., Ann Arbor, MI 48109-2214 USA; 2 NASA Johnson Space Center, Houston, TX 77058 USA; 3 KBRwyle, Houston, TX 77058 USA; 4 Department of Psychology, University of Michigan, Ann Arbor, MI 48109 USA

**Erratum to**: *npj Microgravity (2016)*; doi: 10.1038/s41526-016-0001-9 (2017); Published online 19 December 2016

In this Article, data within Supplementary Figure 1 inadvertently included information that breached subject confidentiality. This information has been removed from Supplementary Figure 1 in the online versions of the paper.

