# Supplementary figures and images for: Brain structural plasticity with spaceflight
Source: NPJ Microgravity. 2016 Dec 19;2:2. doi: 10.1038/s41526-016-0001-9 (PMC5460234; doi:10.1038/s41526-016-0001-9)

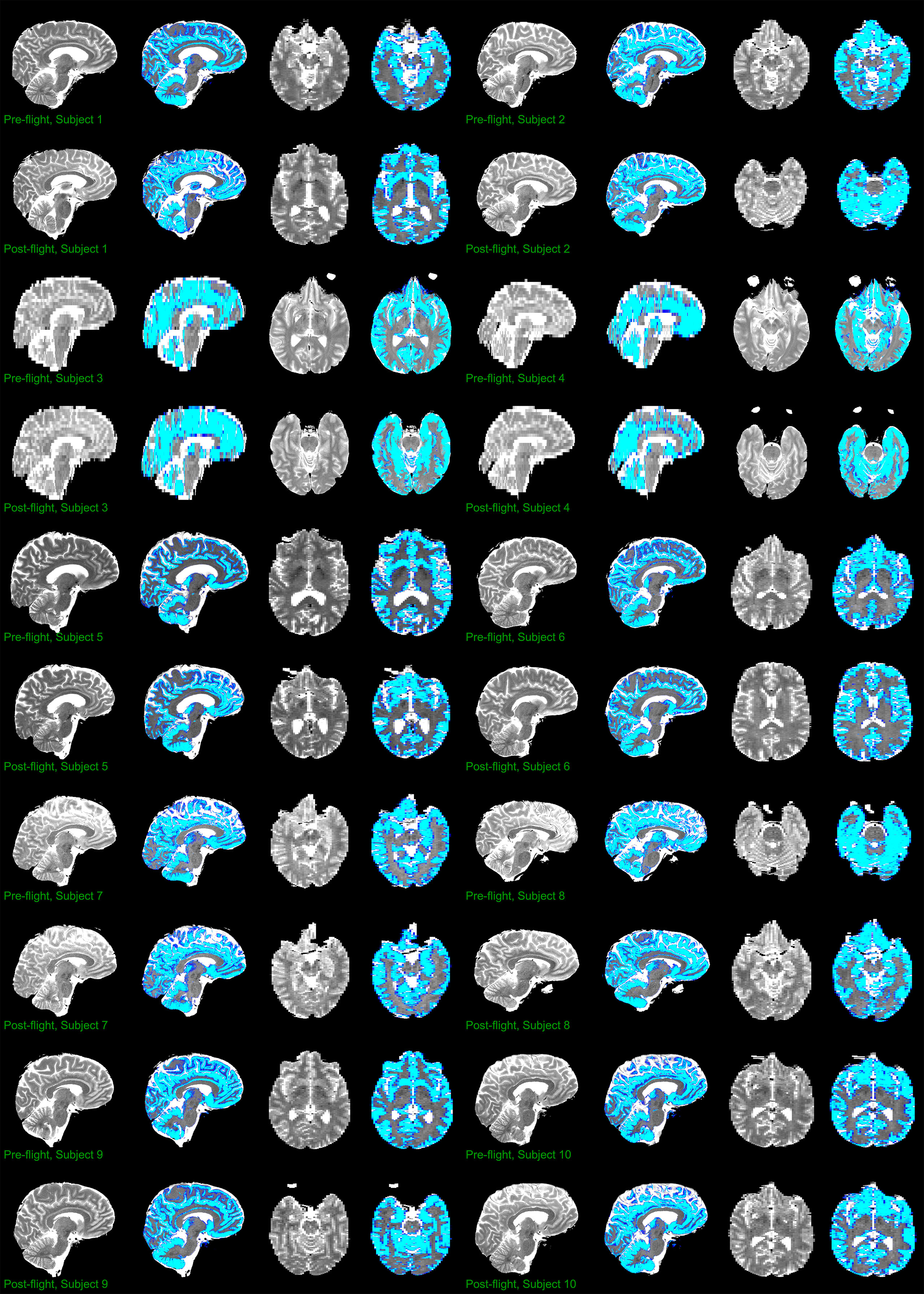

Supplement: Supplementary file 1 — Supplementary Figure 1 [file 41526_2016_1_MOESM1_ESM.jpg]
